# Dysbindin Deficiency Modifies the Expression of GABA Neuron and Ion Permeation Transcripts in the Developing Hippocampus

**DOI:** 10.3389/fgene.2017.00028

**Published:** 2017-03-10

**Authors:** Jennifer Larimore, Stephanie A. Zlatic, Miranda Arnold, Kaela S. Singleton, Rebecca Cross, Hannah Rudolph, Martha V. Bruegge, Andrea Sweetman, Cecilia Garza, Eli Whisnant, Victor Faundez

**Affiliations:** ^1^Department of Biology, Agnes-Scott College,Decatur, GA, USA; ^2^Department of Cell Biology, Emory University,Atlanta, GA, USA

**Keywords:** dysbindin, GABA, parvalbumin, BLOC-1, Neurodevelopmental disorders

## Abstract

The neurodevelopmental factor dysbindin is required for synapse function and GABA interneuron development. Dysbindin protein levels are reduced in the hippocampus of schizophrenia patients. Mouse dysbindin genetic defects and other mouse models of neurodevelopmental disorders share defective GABAergic neurotransmission and, in several instances, a loss of parvalbumin-positive interneuron phenotypes. This suggests that mechanisms downstream of dysbindin deficiency, such as those affecting GABA interneurons, could inform pathways contributing to or ameliorating diverse neurodevelopmental disorders. Here we define the transcriptome of developing wild type and dysbindin null *Bloc1s8^sdy/sdy^* mouse hippocampus in order to identify mechanisms downstream dysbindin defects. The dysbindin mutant transcriptome revealed previously reported GABA parvalbumin interneuron defects. However, the *Bloc1s8^sdy/sdy^* transcriptome additionally uncovered changes in the expression of molecules controlling cellular excitability such as the cation-chloride cotransporters NKCC1, KCC2, and NCKX2 as well as the potassium channel subunits Kcne2 and Kcnj13. Our results suggest that dysbindin deficiency phenotypes, such as GABAergic defects, are modulated by the expression of molecules controlling the magnitude and cadence of neuronal excitability.

## Introduction

Dysbindin is a neurodevelopmental gene product encoded by *DTNBP1*, a gene whose polymorphisms influence cognitive and neuroanatomical traits in non-disease individuals. (Straub et al., 2002; Van Den Bogaert et al., 2003; Bray et al., 2005; Luciano et al., 2009; Markov et al., 2009, 2010; Mechelli et al., 2010; Cerasa et al., 2011; Tognin et al., 2011; Wolf et al., 2011; Ayalew et al., 2012; Trost et al., 2013). *DTNBP1* polymorphisms have been considered risk factors for schizophrenia onset (Straub et al., 2002; Van Den Bogaert et al., 2003), yet this is not a consensus view (Schizophrenia Working Group of the Psychiatric Genomics Consortium, 2014; Farrell et al., 2015). Dysbindin polypeptide expression is reduced in the brain of schizophrenia affected individuals, in particular synaptic fields of the hippocampal formation suggesting a dysbindin requirement for synapse function (Talbot et al., 2004, 2006, 2011; Tang et al., 2009). Dysbindin’s necessity for normal synapse architecture and function has been best documented in dysbindin mutant organisms. The *sandy* mouse, a dysbindin null mutation (*Bloc1s8^sdy/sdy^*), affects the biogenesis of synaptic vesicles and impairs glutamatergic and GABAergic neurotransmission (Jentsch et al., 2009; Ji et al., 2009; Carlson et al., 2011; Ghiani and Dell’angelica, 2011; Karlsgodt et al., 2011; Larimore et al., 2011; Mullin et al., 2011; Yuan et al., 2016). Similarly, *Drosophila* dysbindin mutants are characterized by impaired neurotransmission, abrogated synaptic homeostasis, pre- and post-synaptic morphological alterations, and defects in short term memory (Dickman and Davis, 2009; Cheli et al., 2010; Shao et al., 2011; Dickman et al., 2012; Gokhale et al., 2015a, 2016; Mullin et al., 2015). The reduction of dysbindin in the hippocampus of patients with schizophrenia, alterations in excitatory/inhibitory signaling in the mouse, and changes in neurotransmission impacting short term memory in *Drosophila* demonstrate that dysbindin-dependent pathways provide insight into mechanisms of schizophrenia and other neurodevelopmental disorders. The focus of this work is the characterization of these dysbindin-dependent mechanisms and pathways in the developing mouse brain.

Dysbindin *Bloc1s8^sdy/sdy^* mutant possesses impaired GABAergic neurotransmission, a consequence of decreased parvalbumin positive interneurons (Ji et al., 2009; Carlson et al., 2011; Larimore et al., 2014; Yuan et al., 2016). Similarly, GABA neurotransmission dysfunction has been implicated in multiple neurodevelopmental disorders including autism and schizophrenia (Akbarian et al., 1995; Guidotti et al., 2000; Hashimoto et al., 2003, 2005, 2008a,b; Tabuchi et al., 2007; Gogolla et al., 2009; Sohal et al., 2009; Chao et al., 2010; Han et al., 2012; Marin, 2012; Del Pino et al., 2013; Gonzalez-Burgos et al., 2015; Mariani et al., 2015; Wohr et al., 2015). The convergence on GABA interneuron defects among multiple models of neurodevelopmental disorders, including dysbindin mutants, prompted us to interrogate transcriptional responses of developing hippocampal neurons bearing null mutations in dysbindin. We reasoned mechanisms sensitive to dysbindin mutations would inform us about GABA response pathways implicated in diverse neurodevelopmental disorders. Here we describe transcript modifications in the developing *Bloc1s8^sdy/sdy^* hippocampus. The dysbindin deficiency transcriptome not only captured the previously described GABA interneuron phenotype but, in addition, revealed changes in the expression of molecules controlling cellular excitability such as cation-chloride cotransporters and potassium channel subunits. Our results suggest that GABAergic phenotypes in dysbindin deficiency are developmentally modulated by complex changes in the expression of channels and transporters controlling the magnitude and tempo of neuronal excitability.

## Results

### Null Alleles of the BLOC-1 Subunits Dysbindin, Muted, and Pallid Differentially Affect GABAergic Interneurons

Dysbindin (Bloc1s8) is a subunit of the cytosolic hetero-octamer known as the biogenesis of lysosome-related organelles complex 1 (BLOC-1). This complex consists of Bloc1s1–8 subunits (Li et al., 2003; Wei, 2006; Ghiani et al., 2009; Ghiani and Dell’angelica, 2011). Dysbindin-null *Bloc1s8^sdy/sdy^* mouse hippocampi have reduced numbers of GABAergic interneurons and diverse interneuron markers resulting in impaired inhibitory neurotransmission in the hippocampus (Carlson et al., 2011; Larimore et al., 2014). Here, we explored the ontological and anatomical penetrance of interneuron phenotypes in mouse mutants affecting three subunits of the dysbindin-BLOC-1 complex: dysbindin, muted, and pallid; which are encoded by the genes *Bloc1s8*, *Bloc1s5*, and *Bloc1s6*, respectively, in mouse (Larimore et al., 2014). As a first step, we sought to identify dysbindin-BLOC-1 complex subunit mutant mouse age and anatomical region with the most prominent interneuron phenotypes. We then used this information to identify transcriptional responses to dysbindin-BLOC-1 dependent impaired inhibitory neurotransmission.

**FIGURE 1:**
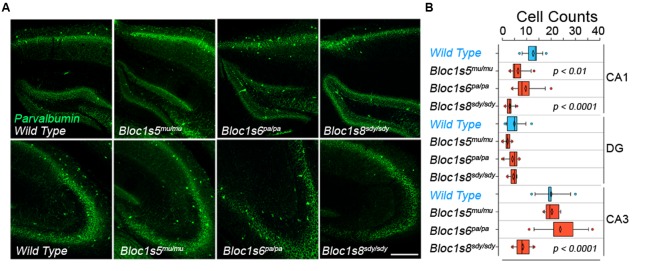
**Differential Effects of BLOC-1 Complex Null Mutations in the Number of Parvalbumin-Positive Neurons.** Hippocampal sections from wild type, *Bloc1s8^sdy/sdy^*, *Bloc1s5^mu/mu^*, and *Bloc1s6^pa/pa^* postnatal day 50 adults were stained with antibodies against parvalbumin and imaged by confocal microscopy. **(A)** Depicts sections of the dentate gyrus and CA1 (upper panels) and the CA3 area (bottom panels) for four genotypes. **(B)** Depicts quantitation of the number of parvalbumin positive cells per section in each one of the genotypes. Cell counts were performed blind to the genotype of the animal. Wild type (*n* = 9), *Bloc1s8^sdy/sdy^* (*n* = 7), *Bloc1s5^mu/mu^* (*n* = 7), and *Bloc1s6^pa/pa^* (*n* = 6) hippocampi analyzed. One Way ANOVA followed by Dunnett’s Multiple Comparison.

We performed quantitative immunofluorescence microscopy of parvalbumin-positive interneurons in the hippocampal formation of adult (P50) wild type and dysbindin-BLOC-1 null mice: *Bloc1s8^sdy/sdy^*, *Bloc1s5^mu/mu^*, and *Bloc1s6^pa/pa^* (**Figure 1A**). We focused on parvalbumin positive cells because of their abundance in hippocampus as compared to other interneuron types (Celio, 1986; Tamamaki et al., 2003; Whissell et al., 2015). In addition, parvalbumin positive interneurons phenotypes in adult P50 *Bloc1s8^sdy/sdy^* hippocampus are shared with other interneurons, which we previously scored with diverse GABAergic transcript markers (Larimore et al., 2014). We determined the numbers of parvalbumin-positive cells in the dentate gyrus, CA1, and CA3 regions of the hippocampus and confirmed previously described decreases in CA1 and CA3 parvalbumin-positive cells in *Bloc1s8^sdy/sdy^* (**Figure 1B**) (Carlson et al., 2011). These results are also in agreement with reduced levels of diverse GABAergic interneuron mRNAs in adult *Bloc1s8^sdy/sdy^* hippocampus (Larimore et al., 2014). In contrast, parvalbumin cell count phenotypes were less pronounced or absent from the CA1 and CA3 regions of *Bloc1s5^mu/mu^* and *Bloc1s6^pa/pa^* (**Figures 1A,B**). We did not detect changes in parvalbumin cell counts in the dentate gyrus in any of the dysbindin-BLOC-1 mutant genotypes analyzed (**Figure 1B**). These results indicate that the most severe and anatomically penetrant GABAergic phenotypes are observed in *Bloc1s8^sdy/sdy^*.

**FIGURE 2:**
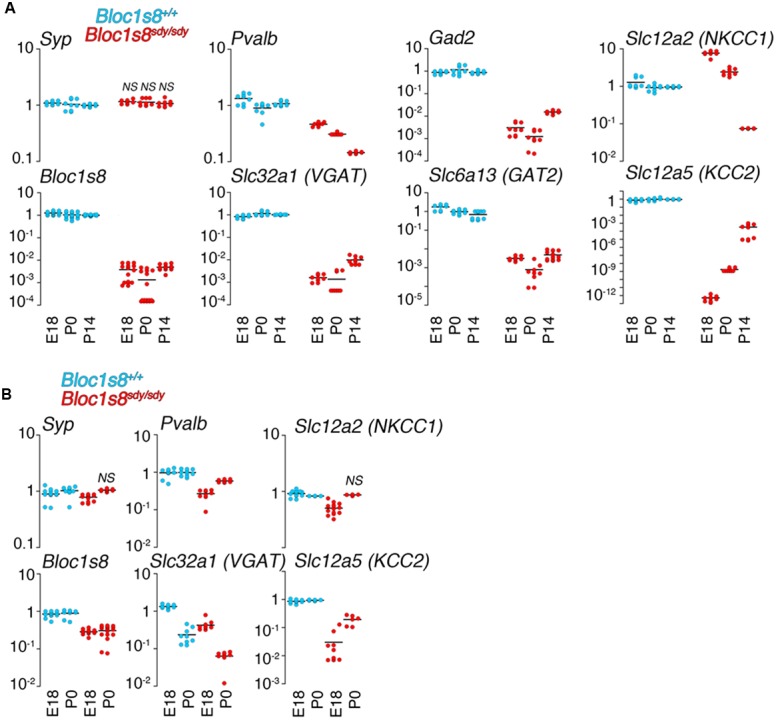
**Developmental Changes in the Expression of GABA Neuron Markers and Chloride Cotransporters Transcripts in Dysbindin Null Hippocampus. (A)** mRNA was isolated from wild type and *Bloc1s8^sdy/sdy^* embryonic day 18, newborn (P0), and postnatal day 14 (P14) hippocampi. Transcripts were quantified by real time quantitative PCR for the indicated GABA neuron gene products and the chloride transporters NKCC1 and KCC2. **(B)** Shows a similar analysis performed in prefrontal cortex. Tissue collections and processing were performed for each age independently, thus qRT-PC comparisons were performed within a specific age. Wild type values for a transcript were defined as 1 at each specified age. All determinations were performed from at least three animals per genotype and two independent cDNA preparations for qRT-PCR. Comparisons between wild type and *Bloc1s8^sdy/sdy^* were performed with Wilcoxon–Mann–Whitney Rank Sum Test. All comparisons were significantly different with a *p* < 0.00041 unless specified otherwise as not significant (NS).

In order to define the ontology and anatomical penetrance of the *Bloc1s8^sdy/sdy^* parvalbumin phenotype, we used quantitative RT-PCR to measure transcripts encoding GABAergic interneuron markers parvalbumin (*Pvalb*), the vesicular GABA transporter (VGAT, *Slc32a1*), the plasma membrane GABA transporter GAT2 (*Slc6a13*), and glutamate decarboxylase 2 (*Gad2*), an enzyme catalyzing the production of gamma-aminobutyric acid from L-glutamic acid (**Figure 2**). mRNA levels of all of these GABA interneurons markers were significantly decreased in the hippocampal formation from embryonic day 18 to 14 days post-natal age (**Figure 2A**). The selectivity of these mRNA modifications was determined by measuring the transcript of synaptophysin (*Syp*), a globally expressed synaptic vesicle protein whose transcripts are insensitive to *Bloc1s8^sdy/sdy^* mutation (Larimore et al., 2014). We reasoned that reductions in GABAergic interneuron function would be reflected in possible compensatory postsynaptic changes in the transcripts of chloride channels that control the inhibitory or excitatory responses of target neurons. NKCC1 and KCC2 are sodium-potassium and potassium-chloride co-transporters, respectively, whose expression levels determine whether GABA induces depolarization or hyperpolarization of target cells (Kaila et al., 2014). Normally, NKCC1 predominates in immature target neurons whereas KCC2 expression is low in immature cells (Kaila et al., 2014). This NKCC1 and KCC2 pattern of expression was drastically accentuated in embryonic 18 and newborn *Bloc1s8^sdy/sdy^* mouse hippocampus (**Figure 2A**). NKCC1 transcripts were increased two- to sixfold in E18 and newborn *Bloc1s8^sdy/sdy^* hippocampus. In contrast, the expression of KCC2 was reduced by several orders of magnitude in *Bloc1s8^sdy/sdy^* hippocampus as compared to age matched wild type animals (**Figure 2A**). These *Bloc1s8^sdy/sdy^* NKCC1 and KCC2 expression trends were reverted (NKCC1) or ameliorated (KCC2) by postnatal day 14, a developmental time where the chloride electrochemical equilibrium potential reaches adult levels (**Figure 2A**) (Berglund et al., 2006; Kaila et al., 2014). We compared these hippocampal GABA neuron marker and chloride transporter mRNA phenotypes with expression of these markers in the prefrontal cortex of embryonic 18 and newborn wild type and *Bloc1s8^sdy/sdy^* (**Figure 2B**). Prefrontal cortex has decreased parvalbumin, VGAT, and KCC2 mRNA levels yet the magnitude of these changes was modest as compared to the mutant hippocampus (**Figure 2B**). These data argue that the most penetrant GABAergic molecular phenotypes occur in the hippocampus of *Bloc1s8^sdy/sdy^* between birth and postnatal age 14. Furthermore, these *Bloc1s8^sdy/sdy^* GABAergic defects associate with changes in the expression of postsynaptic chloride channels that determine GABA-dependent excitatory or inhibitory tone during development.

### Transcriptome Analysis of *Bloc1s8^sdy/sdy^* Hippocampus

**FIGURE 3:**
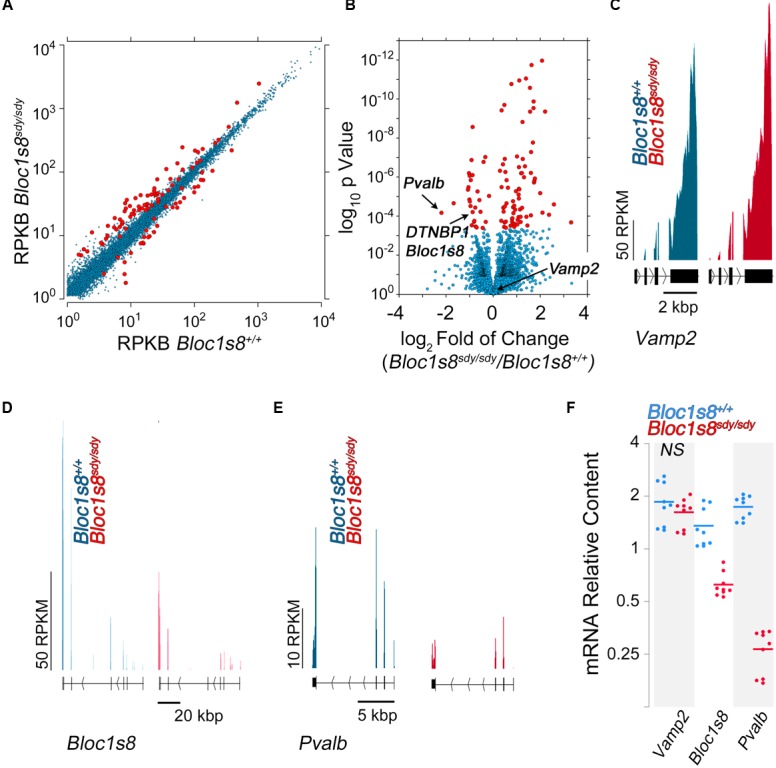
**Transcriptome of the Dysbindin Null Developing Hippocampus. (A)** Scatter plot of normalized read counts per gene calculated by Cuffdiff analysis in postnatal seven hippocampi from wild type and *Bloc1s8^sdy/sdy^* animals, *n* = 3. RKBP = reads per kilobase of transcript per million mapped reads. Red dots represent genes differentially expressed with a *q*-value <0.05. **(B)** Volcano plot of Cuffdiff analysis shows (Trapnell et al., 2012) differentially expressed genes in *Bloc1s8^sdy/sdy^* hippocampus as in **(A)**. Red dots correspond to genes with >2 log_2_-fold differential expression or <-1 log_2_-fold differential expression. **(C–E)** Illumina sequence reads maps for the listed mouse genes from assembly mm9. VAMP2 is a control gene whose expression is not sensitive to *Bloc1s8^sdy/sdy^*. **(F)** Postnatal day 7 hippocampi were quantified by real time quantitative PCR for the indicated gene products. All determinations were performed from at least three animals per genotype and three independent cDNA preparations for qRT-PCR. Comparisons between wild type and *Bloc1s8^sdy/sdy^* were performed with Wilcoxon–Mann–Whitney Rank Sum Test. All comparisons were significantly different with a *p* < 0.0005 unless specified otherwise as not significant (NS).

We performed RNAseq of the hippocampus of wild type and *Bloc1s8^sdy/sdy^* hippocampus at postnatal day seven (**Figure 3** and **Supplementary Table S1**). We selected this age because it is in between the ages with the most pronounced GABAergic transcript levels modifications (**Figure 2**), and it coincides with peak hippocampal synaptogenesis in mice (Mody et al., 2001). We prepared normalized polyA cDNA libraries of three wild type and three *Bloc1s8^sdy/sdy^* hippocampi and determined the abundance of transcripts by Illumina HiSeq2000 sequencing (Mortazavi et al., 2008). Illumina sequencing quantitation identified 111 transcripts whose content was significantly modified in *Bloc1s8^sdy/sdy^* hippocampi (*q* < 0.005, see **Supplementary Table S1**). Eighty of the transcripts were upregulated and 31 were downregulated (**Figures 3A,B** and **Supplementary Table S1**). The transcriptome did not detect the changes in expression of NKCC1 or KCC2 detected by qRT-PCR (compare **Figure 2** and **Supplementary Table S1**). However, the transcriptome identified landmark mRNAs that validated the transcript dataset. First, we found that transcripts encoding dysbindin (*DTNBP1, Bloc1s8*) and parvalbumin (*Pvalb*) were reduced in *Bloc1s8^sdy/sdy^* hippocampi by 50% (**Figures 3B,D,E** and **Supplementary Table S1**). Additionally, transcripts of synaptic vesicle proteins insensitive to the *Bloc1s8^sdy/sdy^* mutation and used as controls, such as synaptophysin and VAMP2, were not affected by the *Bloc1s8^sdy/sdy^* mutation (**Figures 3B,C** and **Supplementary Table S1**) (Larimore et al., 2014). We confirmed mRNA expression of all of these markers by qRT-PCR in wild type and *Bloc1s8^sdy/sdy^* seven day postnatal hippocampi (**Figure 3F**).

**FIGURE 4:**
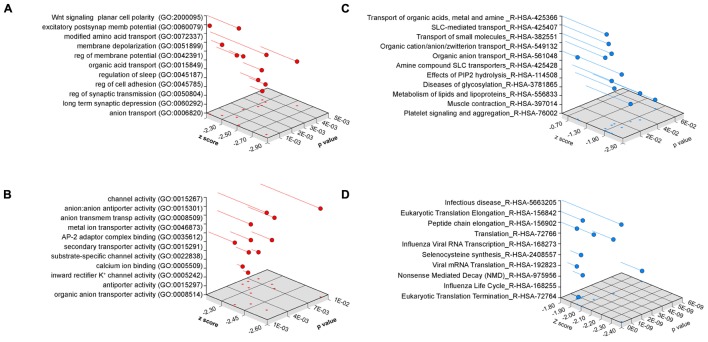
**Ontology Analysis of the Dysbindin Null Developing Hippocampus.** Gene ontology analysis of the *Bloc1s8^sdy/sdy^* P7 hippocampus using ENRICHR for **(A)** biological process, **(B)** molecular function, and **(C)** the REACTOME database. **(D)** Depicts ENRICHR REACTOME analysis for a custom generated random human brain gene data set presented in **Supplementary Table S2**.

We performed gene ontology analysis to prioritize transcripts associated to pathways and mechanisms for independent confirmation and further study (**Figure 4**). We used two different bioinformatics algorithms, ENRICHR and DAVID, to statistically assess gene product enrichment within gene ontological (GO) terms (Huang da et al., 2009; Chen et al., 2013). The major biological process GO terms associated to the *Bloc1s8^sdy/sdy^*-sensitive transcriptome were Wnt signaling planar cell polarity (BP GO:2000095, *p* < 0.00006), excitatory postsynaptic membrane potential (BP GO:0060079, *p* < 0.00002) as well as ontological terms associated to solute transport and ion permeation through membranes (all *p*-values <0.0036, **Figure 4A**). Similar outcomes were obtained either by analyzing molecular function GO terms where the top term was channel activity (MF GO:0015267, *p* < 0.0027, **Figure 4B**) or by using the REACTOME database, where top terms where implicated in solute transport (R-HSA-425366 and R-HSA-425407, *p-*values <0.000032 and <0.00017, respectively, **Figure 4C**). Gene ontology analysis using the DAVID algorithm lent the same results as ENRICHR. Ion transport emerged as the top biological process category (BP GO:0006811 *p* < 4.29E-04). Gene ontology categories identified in the *Bloc1s8^sdy/sdy^*-sensitive transcriptome were not recognized within a random brain gene dataset of comparable size (**Figure 4D**). Similarly, simultaneous ontology analyses of the random brain gene data set and the *Bloc1s8^sdy/sdy^*-sensitive transcriptome using the engine GeneCodis showed no ontology overlap (Tabas-Madrid et al., 2012).

**FIGURE 5:**
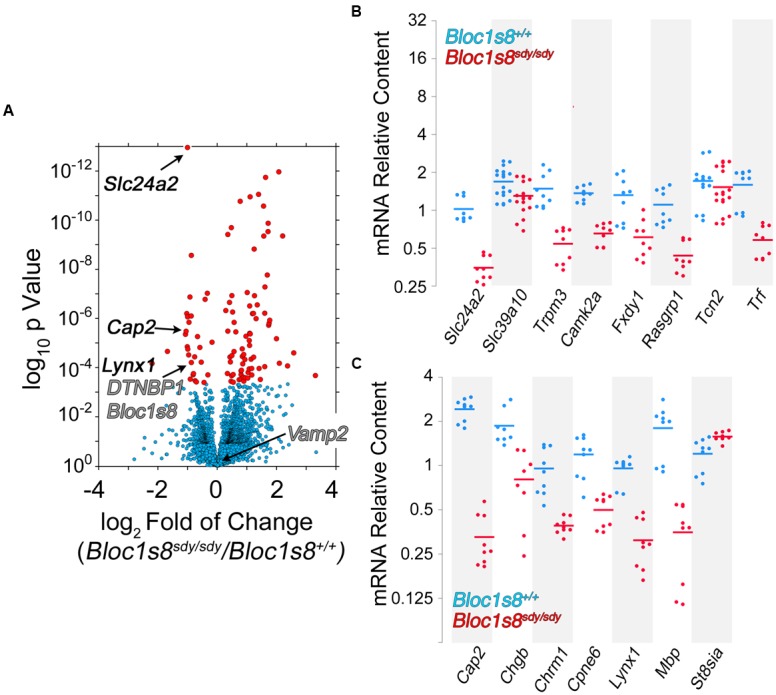
**Dysbindin null developing hippocampus changes the expression of ion transport gene products. (A)** Volcano plot of Cuffdiff analysis (Trapnell et al., 2012) shows differentially expressed genes in *Bloc1s8^sdy/sdy^* hippocampus as in **Figure 3B**. Arrows mark genes belonging the ion transport ontology terms and neurological disorder according to DAVID analysis. **(B,C)** Postnatal day 7 hippocampi mRNA were quantified by real time quantitative PCR for the indicated gene products belonging to ion transport ontology terms **(B)** and neurological disorder **(C)**. All determinations were performed from at least three animals per genotype and three independent cDNA preparations for qRT-PCR. Comparisons between wild type and *Bloc1s8^sdy/sdy^* were performed with Wilcoxon–Mann–Whitney Rank Sum Test. All comparisons were significantly different with a *p* < 0.005.

Twelve percent of the 111 transcripts sensitive to *Bloc1s8^sdy/sdy^* belonged to ion transport gene ontology terms. Among these gene products are those encoded by *Fxyd1*, *Trpm3*, *Slc39a10*, *Slc22a8*, *Slc24a2*, *Slc22a6*, *Slc13a4*, *Tcn2*, *Trf*, *Kcnj13*, *Kcne2*, *Camk2a*, and *Steap1*. We focused on these ion transport gene products to confirm by qRT-PCR the *Bloc1s8^sdy/sdy^* transcriptome dataset. In total, we chose for confirmation by qRT-PCR 19 of the 111 transcriptome hits. Among these confirmed hits were *Fxyd1*, *Trpm3*, *Slc39a10*, *Slc24a2*, *Trf*, *Kcnj13*, *Kcne2*, and *Camk2a*, which belong to the ion transport ontology term (GO:0006811, **Figures 5A,B**). In addition, we confirmed *Cap2*, *Chgb*, *Cpne6*, *Lynx1*, and *Mbp*; which were selected among gene products implicated in schizophrenia and neurological disorders by the DAVID algorithm (*p* < 2.35E-03, **Figure 5C**). These results validate the *Bloc1s8^sdy/sdy^* hippocampal transcriptome dataset by means of an ontological prioritization of mRNA phenotypes.

**FIGURE 6:**
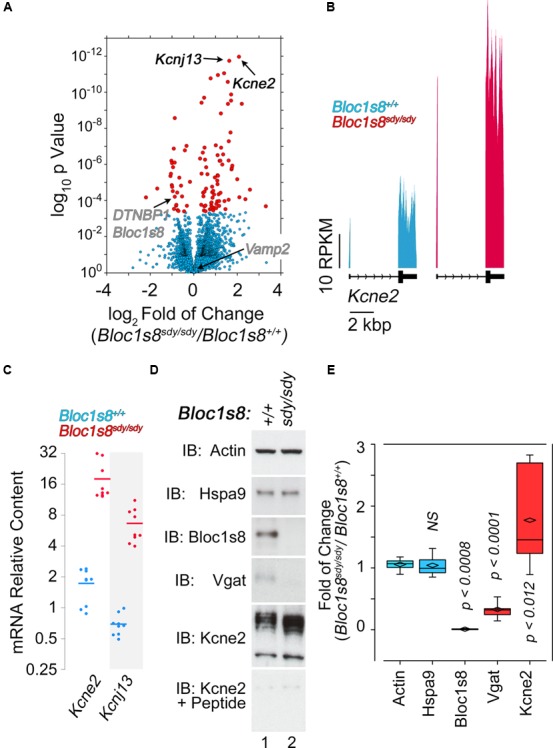
**Dysbindin null developing hippocampus changes the expression of potassium channel subunits. (A)** Volcano plot of Cuffdiff analysis shows (Trapnell et al., 2012) differentially expressed genes in *Bloc1s8^sdy/sdy^* hippocampus as in **Figure 3B**. Arrows mark potassium channel subunit genes for reference. **(B)** Illumina sequence reads maps for the listed genes from assembly mm9. **(C)** Postnatal day 7 hippocampi mRNA were quantified by real time quantitative PCR for *Kcne2* and *Kcnj13*. All determinations were performed from at least three animals per genotype and three independent cDNA preparations for qRT-PCR. Comparisons between wild type and *Bloc1s8^sdy/sdy^* were performed with Wilcoxon–Mann–Whitney Rank Sum Test. All comparisons were significantly different with a *p* < 0.0001. **(D,E)** Immunoblot analysis of Kcne2 expression in wild type and *Bloc1s8^sdy/sdy^* hippocampus P50 adult hippocampus in the absence or presence of the Kcne2 antigenic peptide. Control blots with dysbindin antibodies (Bloc1s8), Vgat and loading controls (actin and Hspa9) are presented. **(E)** Comparisons between actin and other antigens were performed Kruskal–Wallis Rank Sum Test followed by pairwise Wilcoxon–Mann–Whitney Rank Sum Test.

NCKX2, which is encoded by *Slc24a2* is the most significantly reduced transcript in *Bloc1s8^sdy/sdy^* hippocampus (**Figure 5**). NCKX2 is a sodium-potassium-calcium exchanger that regulates calcium transients and excitability (Lee et al., 2002; Jeon et al., 2003; Kim et al., 2005; Stephan et al., 2011). *Kcnj13* and *Kcne2* are the most significantly upregulated transcripts in *Bloc1s8^sdy/sdy^* hippocampus (**Figure 6A**). We focused our studies in these potassium channels as they may represent compensatory mechanisms secondary to impaired GABAergic neurotransmission in *Bloc1s8^sdy/sdy^* brain. Kcnj13 is an inward rectifier potassium channel, also known as Kir7.1, whereas Kcne2 or MiRP1 is a voltage gated potassium channel modulatory subunit. Loss of any one of these two channel subunits results in increased membrane excitability both in neurons and muscle cells (Ying et al., 2012; Abbott et al., 2014; McCloskey et al., 2014). Illumina sequencing digital quantitation of these two transcripts showed that *Kcnj13* increased threefold while Kcne2 increased 4.5-fold in mutant hippocampus as compared to controls (**Figures 6A,B**). We confirmed these *Bloc1s8^sdy/sdy^* mRNA changes by qRT-PCR and found that *Kcnj13* and *Kcne2* increased their expression by 9.6- and 10.5- fold, respectively, in postnatal day seven mutant hippocampus (**Figure 6C**). These changes in mRNA led to increased protein expression. We measured protein levels of Kcne2 polypeptide in postnatal day 50 wild type and mutant hippocampus. We focused on Kcne2 because antibodies recognized bands of the proper molecular weight, which were abolished by preincubating antibodies with the antigenic Kcne2 peptide (**Figures 6D,E**). We used as loading controls actin and the mitochondrial chaperone mortalin (Hspa9) and as positive controls dysbindin (Bloc1s8) and the GABA vesicular transporter (Vgat) (**Figures 6D,E**). Protein levels of Kcne3 increased by two- to threefold in mutant hippocampus (**Figures 6D,E**). Since these immunoblots were performed in young adult hippocampus, postnatal day 50, these results demonstrate that increased expression of a potassium channel subunit that decreases excitability is persistent phenotypes downstream of *Bloc1s8^sdy/sdy^*. We propose that changes in the expression of NKCC1, KCC2, NCKX2, Kcne2, and Kcnj13 represent developmental adaptive responses to a reduced GABAergic tone induced by genetic defects in a neurodevelopmental gene, *Bloc1s8*.

## Discussion

**FIGURE 7:**
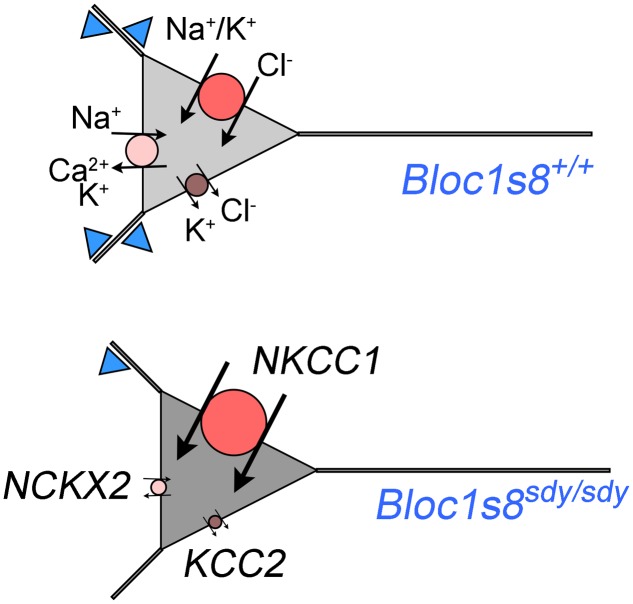
**A model of phenotypes downstream of GABA interneurons in dysbindin null developing hippocampus.** Diagram depicts a model of wild type and *Bloc1s8^sdy/sdy^* neuron innervated by parvalbumin-positive GABA terminals (blue triangles) at postnatal day 7. The *Bloc1s8^sdy/sdy^* mutation decreases the number of GABA cells and synapses (reduced blue triangles). Changes in the expression of NKCC1, KCC2, and NCKX2 are represented by the size of the circle. Arrows depict direction of ion flows. The shade of gray represents predicted intracellular chloride concentration, note an increased concentration of intracellular chloride in *Bloc1s8^sdy/sdy^* neurons due to changes in expression in transporters expression.

We described GABA neuron defects and transcriptional responses downstream of a mutation in a neurodevelopmental gene product, dysbindin, encoded by *DTNBP1* and *Bloc1s8* in human and mice, respectively. We identified two novel molecular phenotypes in dysbindin deficiency (**Figure 7**). First, we found drastic changes in the expression of the cation-chloride cotranspoters NKCC1 and KCC2 during hippocampal development. These NKCC1 and KCC2 expression ratio changes are similar to those described in schizophrenia patient brains where the NKCC1 and KCC2 expression ratio is increased (Hyde et al., 2011; Sullivan et al., 2015). Second, the transcriptome of *Bloc1s8^sdy/sdy^* postnatal day 7 hippocampus is enriched in transcripts implicated in ionic regulation. In particular, one of the most prominently downregulated messages was SLC24A2 which encodes the sodium-potassium-calcium exchanger 2 or NCKX2. Loss of NCKX2 protein or its transport function increases cytoplasmic calcium levels, thus prolonging the tempo of cellular excitability (Lee et al., 2002; Jeon et al., 2003; Kim et al., 2005; Stephan et al., 2011). In contrast, we found that two of the most upregulated messages in *Bloc1s8^sdy/sdy^* hippocampus encode potassium channel subunits Kcne2 and Kcnj13. We documented increased expression of the message and protein expression of Kcne2, an ancillary regulatory potassium channel subunit in the hippocampus of postnatal day 7 and adult *Bloc1s8^sdy/sdy^* animals. Loss of *Kcne2* or *Kcnj13* increase cellular excitability (Ying et al., 2012; Abbott et al., 2014; McCloskey et al., 2014), thus our findings suggest that their overexpression in *Bloc1s8^sdy/sdy^* hippocampus may decrease cellular excitability.

The transcriptome of *Bloc1s8^sdy/sdy^* hippocampus identified reduced expression of *Bloc1s8*, *Pvalb*, and 111 additional transcripts, yet it did not detect modifications in the expression of NKCC1 and KCC2 transcripts documented here by qRT-PCR. Similarly, the transcriptome did not detect changes in the expression of the copper homeostasis molecules *Atp7a*, *Atox1*, and *Lox*; as well as other GABA neuron markers previously documented by us (Larimore et al., 2014; Gokhale et al., 2015b). This argues that our transcriptome either was not sufficiently powered to identify all transcripts sensitive to the *Bloc1s8^sdy/sdy^* mutation in the developing hippocampus or the depth of library sequencing was insufficient. However, the transcriptome still illuminated new ion channel and transport mechanisms secondary to the *Bloc1s8^sdy/sdy^* mutation. A compromised discovery of transcripts sensitive to the *Bloc1s8^sdy/sdy^* allele may be a contributing factor to explain why the transcriptome gene ontology poorly overlaps with the ontology of the *Bloc1s8^sdy/sdy^* and the BLOC-1-sensitive proteomes (Gokhale et al., 2016). An additional factor to account for the poor overlap between ontologies of the proteome and transcriptome in *Bloc1s8^sdy/sdy^* genetic defects is the relative protein abundance of channels and transporters as compared to other proteins. For example, beta actin is present in 114 million copies per HeLa cell. In contrast, NKCC1 is present in ∼100,000 copies per HeLa cell while the Arp2/3 complex is present in 2 million copies per cell (Itzhak et al., 2016). We found that the Arp2/3 complex is downregulated by proteomics in dysbindin mutations and that the Arp2/3 complex genetically and biochemically interacts with dysbindin (Gokhale et al., 2016). Thus, the *Bloc1s8^sdy/sdy^* transcriptome likely contributes hits that escape the partial coverage and comparative sensitivity afforded by proteomic studies. It is interesting that adding the *Bloc1s8^sdy/sdy^* transcriptome hits to the BLOC-1 sensitive proteome hits minimally modifies the gene ontology terms associated to the BLOC-1 sensitive proteome (**Supplementary Figure S1**). In contrast, ionic channel ontology terms discovered with the *Bloc1s8^sdy/sdy^* sensitive transcriptome are no longer top priority when added to the BLOC-1 sensitive proteome. Thus, proteome and transcriptome gene ontologies associated to *Bloc1s8^sdy/sdy^* are complementary in the identification of mechanisms that require dysbindin or compensate *Bloc1s8* genetic defects.

How can we reconcile that loss of dysbindin during development modifies the expression of molecules with apparent opposite roles in excitability? On one hand, the *Bloc1s8^sdy/sdy^* mutation would increase neuronal excitability by changes in NKCC1, KCC2, and NCKX2 expression (**Figure 7**). On the other hand, dysbindin mutation would increase the expression of potassium channel subunits (Kcne2 and Kcnj13), which would decrease neuronal excitability. We favor a model where pro-excitatory changes in the expression of NKCC1, KCC2, and NCKX2 would be confined to a cell type in the developing hippocampus distinct from cells where increased expression Kcne2 and Kcnj13 occurs (**Figure 7**). Increase expression of the potassium channel ancillary subunit Kcne2 is predicted to decrease neuronal excitability based on the functional consequences of *Kcne2* mutation in excitable tissues (Ying et al., 2012; Abbott et al., 2014; McCloskey et al., 2014). Mutations in *KCNE2* in human heart causes a long QT-syndrome and ventricular fibrillation by diminishing potassium currents while mouse null mutations in *Kcne2* increase the excitability of cortical pyramidal neurons (Abbott et al., 1999, 2014). However, the function of Kcne potassium channel subunits is more nuanced. Overexpression of Kcne2 could influence diverse potassium channels at the level of gating, selectivity, conductivity, and channel traffic such as their movement along the exocytic route, endocytosis, and channel polarized cell distribution. (McCrossan and Abbott, 2004; Kanda et al., 2011a,b,c; Abbott, 2015). Some of these properties associated to Kcne2 family members could occur in the same cells where the expression of NKCC1 and KCC2 is modified as a way to modulate the frequency of GABA excitatory potentials in developing hippocampus.

Increased NKCC1, and decreased KCC2 and NCKX2 expression changes in *Bloc1s8^sdy/sdy^* hippocampus are more pronounced between post-natal days 0 to 7, a time where GABA neurotransmission is excitatory due to high intracellular concentration of chloride (Berglund et al., 2006; Ben-Ari et al., 2007; Kaila et al., 2014). We speculate that the increased expression of NKCC1 and the drastically reduced expression of KCC2 would increase intracellular chloride concentrations in developing P7 hippocampal *Bloc1s8^sdy/sdy^* neurons (**Figure 7**). An increased intracellular chloride concentration would in turn compensate the reduction in parvalbumin-positive cells by increasing depolarizing responses to GABA neurotransmission in *Bloc1s8^sdy/sdy^* hippocampus (**Figure 7**). Although this model is speculative, we believe it is of importance. The function of NKCC1 and/or KCC2 can be inhibited by the FDA approved drugs bumetanide and furosemide, respectively (Loscher et al., 2013). Bumetamide has been used to revert persistently increased intracellular chloride concentrations in a mouse model of Down syndrome and improve cognitive outcomes (Deidda et al., 2015). This observation raises the prospect of NKCC1 inhibitors as developmental modulators of GABAergic neurotransmission in *Bloc1s8^sdy/sdy^* and other disorders of GABAergic neurotransmission such as schizophrenia. However, if as we postulate here, increased NKCC1 expression in the developing *Bloc1s8^sdy/sdy^* hippocampus is a compensatory mechanism for reduced GABAergic innervation, then bumetanide may be ineffective or have deleterious effects in *Bloc1s8^sdy/sdy^* and other neurodevelopmental genetic defects affecting GABA neurotransmission such as schizophrenia. In fact, chronic inhibition of NKCC1 with bumetanide during development induces endophenotypes that resemble those in schizophrenia and the *Bloc1s8^sdy/sdy^* mutation (Wang and Kriegstein, 2011). Bumetanide is ineffective in treating positive and negative symptoms in schizophrenia patients (Rahmanzadeh et al., 2016). However, inhibition of KCC2 during development may be an effective strategy to ameliorate neurodevelopmental disorders with impaired GABA neurotransmission. Our findings indicate that the GABA neurotransmission defects observed in *Bloc1s8^sdy/sdy^* mice offer a model to study the functional and anatomical consequences of modulating GABA neurotransmission and cellular excitability by pharmacological agents that target potassium channels and cation transporters that modulate intracellular chloride and calcium concentration.

## Materials and Methods

### Antibodies

Antibodies utilized in this study: rabbit anti-parvalbumin (ThermoFisher Scientific PA1-933), mouse anti-synaptophysin (EMD Millipore MAB5258), mouse anti-actin-beta (Sigma A5451), mouse anti-Hspa9/mortalin (NeuroMab N52A/42), rabbit anti-Bloc1s8 (gift from Dr. Talbot), mouse anti-Vgat (Synaptic Systems 131011), and rabbit anti-Kcne2 (Alomone APC-054).

#### Animals and Tissue Preparation

Mice null for dysbindin (*Bloc1s8^sdy/sdy^*), muted (*Bloc1s5^mu/mu^*) and pallidin (*Bloc1s6^pa/pa^*) were previously described (Larimore et al., 2014). Mice were bred in-house following IUCAC approved protocols. All animals were in in the C57/Black6 background

### qRT-PCR

Hippocampal regions were dissected from E18 to P14 and young adult animals between P48-P52 then flash frozen. mRNA was isolated using TRIzol (Invitrogen Life Technologies, Grand Island, NY, USA) extraction and then reverse transcribed into a cDNA using Super Script III First-Strand Synthesis (Invitrogen Life Technologies, Grand Island, NY, USA). Quantitative PCR amplifications were performed on a LightCycler480 Real Time plate reader using Light Cycler 480 SYBR Green reagents (Roche, Indianapolis, IN) at 95°C for 5 min followed by 45 cycles of 95°C for 5 s, 65°C for 10 s, and 72°C for 20 s followed by a 95°C for 5 s, 65°C for 2 min and a 97°C incubation to determine melting curves. Supplementary Primers Table describes the primers used in this study (**Supplementary Primers Table S1**).

### Immunofluorescence Labeling for Confocal Microscopy

Brain slices were prepared from adult mice at P50 as described (Larimore et al., 2011; Larimore et al., 2013). Following ketamine treatment, animals were transcardially perfused with Ringer’s solution and then perfused with fixative (4% paraformaldehyde with 0.1% glutaraldehyde). For 12-18 h, brains were post-fixed in 4% paraformaldehyde. Following post-fixation, brains were cut into 60 μm thick sections and stored in antifreeze (0.1 M sodium phosphate monobasic, 0.1 M sodium phosphate dibasic heptahydrate, 30% ethylene glycol, 30% glycerol) at –20°C. Brain sections containing the hippocampus were incubated in 1% sodium borohydride. For 60 minutes, tissue was pre-incubated (5% NHS and 1% BSA and 0.3% Triton X-100). Tissue was incubated in primary antibody overnight (anti-Parvalbumin 1:200 with anti-Synaptophysin 1:10,000, 1% NHS and 1% BSA) followed by 60 min in a secondary antibody (1% NHS and 1% BSA 1:500 anti-mouse 568 and anti-rabbit 488) (Invitrogen Molecular Probes, Carlsbad, CA, USA). Finally, tissue was incubated for 30 min in cupric sulfate (3.854 W/V Ammonium Acetate, 1.596 W/V Cupric Sulfate, pH 5). Tissue sections were mounted on slides with Vectashield (Vector Laboratories). Confocal microscopy of immunofluorescent samples was performed with an Anxiovert 100M (Carl Zeiss) coupled to an Argon laser, HeNe1 laser, and Titanium Sapphire laser. Z-stacks were acquired using Plan Apochromat 20X/0.5 dry objective. The emission filters used for fluorescence imaging were BP 505–530 and LP 560. Images were acquired with ZEN (Carl Zeiss). Parvalbumin-positive cells were scored by creating a region of interest (ROI) for each region of the hippocampus. Experimenters were blinded to which genotype was analyzed and the intensity of fluorescence in the section was not taken into account for scoring. Cells were considered parvalbumin-positive if the cell body and dendrites were distinguishable from the surrounding fluorescence in the ROI. More than one blind experimenter scored each genotype and each region. For each experiment, a minimal of four control hippocampi and four null hippocampi were processed independently.

### Immunoblot Analyses

Brain lysates were separated for SDS–PAGE and transferred to PVDF membranes (BioRad, Hercules, CA, USA). Membranes were probed with primary antibodies followed by HRP-conjugated anti-rabbit and anti-mouse secondary antibodies (Santa Cruz Biotechnology, Santa Cruz, CA, USA and Invitrogen). Secondary antibodies were detected using Supersignal West Dura Extended Duration or Western Lightning substrate (Pierce Chemical, Rockford, IL, USA and Perkin Elmer) and developed on film. Quantitation was performed as follows, multiple film exposures were obtained and those films within a liner range were used for quantitation using Fiji (Schindelin et al., 2012). Antigen expression was compared to actin and Hspa9 as housekeeping gene products.

### Transcriptomic and Gene Ontology Analysis

RNAseq services were contracted from Otogenetics Corporation (Atlanta, GA, USA). Briefly, RNA from P7 control and dysbindin mutant hippocampi were extracted with Trizol Reagent and polyA RNA was isolated. The integrity and purity of RNA were determined with Agilent Bioanalyzer. cDNA was prepared with Clontech SmartPCR cDNA kit (Clontech Laboratories). cDNA fragmentation was done with Covaris shearing, profiled with Agilent Bioanalyzer, and subjected to Illumina library preparation with NEB Next reagents (New England Biolabs). RNA content per sample was normalized with ERCC Spike-in (Ambion Thermo Fisher Scientific). The quality, quantity as well as size span of Illumina libraries were determined with an Agilent Bioanalyzer. The libraries were sequenced with Illumina HiSeq2000 sequencing according to standard procedures with a minimum number of 20 million reads per sample. Sequencing data sets from illumina HiSeq2000 were mapped with *Tophat (v2.0.5)* against reference assembly UCSC mm9. Mapping from Tophat were analyzed by *cufflinks.cuffdiff (v.2.0.2)*, to measure expression level (Trapnell et al., 2012). Expression levels were measured with RPKM (Reads Per Kilobase per Million mapped). q-value less than 0.05 was considered as statistically significant. Data are accessible upon request through DNANexus.

We used three algorithms to perform gene ontology analysis: GeneTerm Linker^1^ (Fontanillo et al., 2011), ENRICH^2^ (Chen et al., 2013), and Database Annotation, Visualization and Integrated Discovery^3^ (DAVID) (Huang da et al., 2009). Briefly gene lists uploaded to DAVID were analyzed by setting annotations based by species (Huang da et al., 2009). Parameters for these functional annotation charts included *P*-value cutoff (<0.01), a minimum number of three genes for each GO term, fold enrichment, Bonferroni, and/or Benjamini corrected statistical analysis to control for false discovery (Huang da et al., 2009).

The *Bloc1s8^sdy/sdy^*-sensitive transcriptome was compared against a brain enriched random gene data with ENRICHR algorithm described above. We selected the top 100 genes expressed in hippocampus, caudate, and cortex from http://www.gtexportal.org/home/ (**Supplementary Table S2**) (The GTEx Consortium, 2013, 2015). Three hundred top expressed genes were randomized using the Excel function = RANDBETWEEN(1,n) and the first 150 genes were selected in block for gene ontology analyses. None of the ontology terms in GO BP, MF, and the REACTOME database were common between the *Bloc1s8^sdy/sdy^*-sensitive transcriptome gene dataset and the brain random dataset. We further confirmed the lack of overlap in gene ontology terms between the random brain gene data set and the *Bloc1s8^sdy/sdy^*-sensitive transcriptome with GeneCodis, and engine that allows for the comparison of two datasets simultaneously (Tabas-Madrid et al., 2012).

### Statistical Analysis

Experimental conditions were compared using Synergy Kaleida-Graph, version 4.1.3 (Reading, PA) or Aabel NG2 v5 x64 by Gigawiz as specified in each figure. Transcriptomic data were processed by the *cufflinks.cuffdiff (v.2.0.2)* which corrected for multiple comparisons using Benjamini–Hochberg procedure. These values are expressed as *q*-values in **Supplementary Table S1**. Significance was considered in the transcriptome at a *q* threshold of 0.05 and marked as yes in **Supplementary Table S1**. qRT-PCR results were subjected to tests specified in each figure legend without multiple comparisons corrections.

## Ethics Statement

This study was carried out in accordance with the recommendations of Emory IUCAC. The protocol was approved by the IUCAC.

## Author Contributions

JL, SZ: collected data and edited paper. MA, KS, RC, HR, MB, AS, CG, EW: collected data. VF: designed project, analyzed data, designed figures, wrote the paper.

## Conflict of Interest Statement

The authors declare that the research was conducted in the absence of any commercial or financial relationships that could be construed as a potential conflict of interest.

## Footnotes

1 http://gtlinker.cnb.csic.es/gtset/index

2 http://amp.pharm.mssm.edu/Enrichr/

3 https://david.ncifcrf.gov

## Supplementary Material

FIGURE S1 **Ontology analysis of the BLOC-1 sensitive proteome with or without addition of the *Bloc1s8^sdy/sdy^* hippocampus transcriptome.** Gene ontology analysis of the BLOC-1 sensitive proteome using the Geneterm Linker algorithm was performed with and without addition of the *Bloc1s8^sdy/sdy^* P7 hippocampus transcriptome hits. The numbers in the *Z* axis represent the gene ontology term groups defined by Geneterm Linker as metagroups (Fontanillo et al., 2011). The internal tightness of each metagroup and its separation from other metagroups is defined by the Silhouette score. Strong tightness between terms within a metagroup is defined by a positive Silhouette score.

TABLE S1 **Cufflinks Analysis of Bloc1s8-null Transcriptome**.

TABLE S2 **Brain Enriched Randomized Gene Set**.

PRIMERS TABLE S1 **Primer list for qRT-PCR experiments**.
