# Supplementary figures and images for: Dysbindin Deficiency Modifies the Expression of GABA Neuron and Ion Permeation Transcripts in the Developing Hippocampus
Source: Front Genet. 2017 Mar 10;8:28. doi: 10.3389/fgene.2017.00028 (PMC5344932; doi:10.3389/fgene.2017.00028)

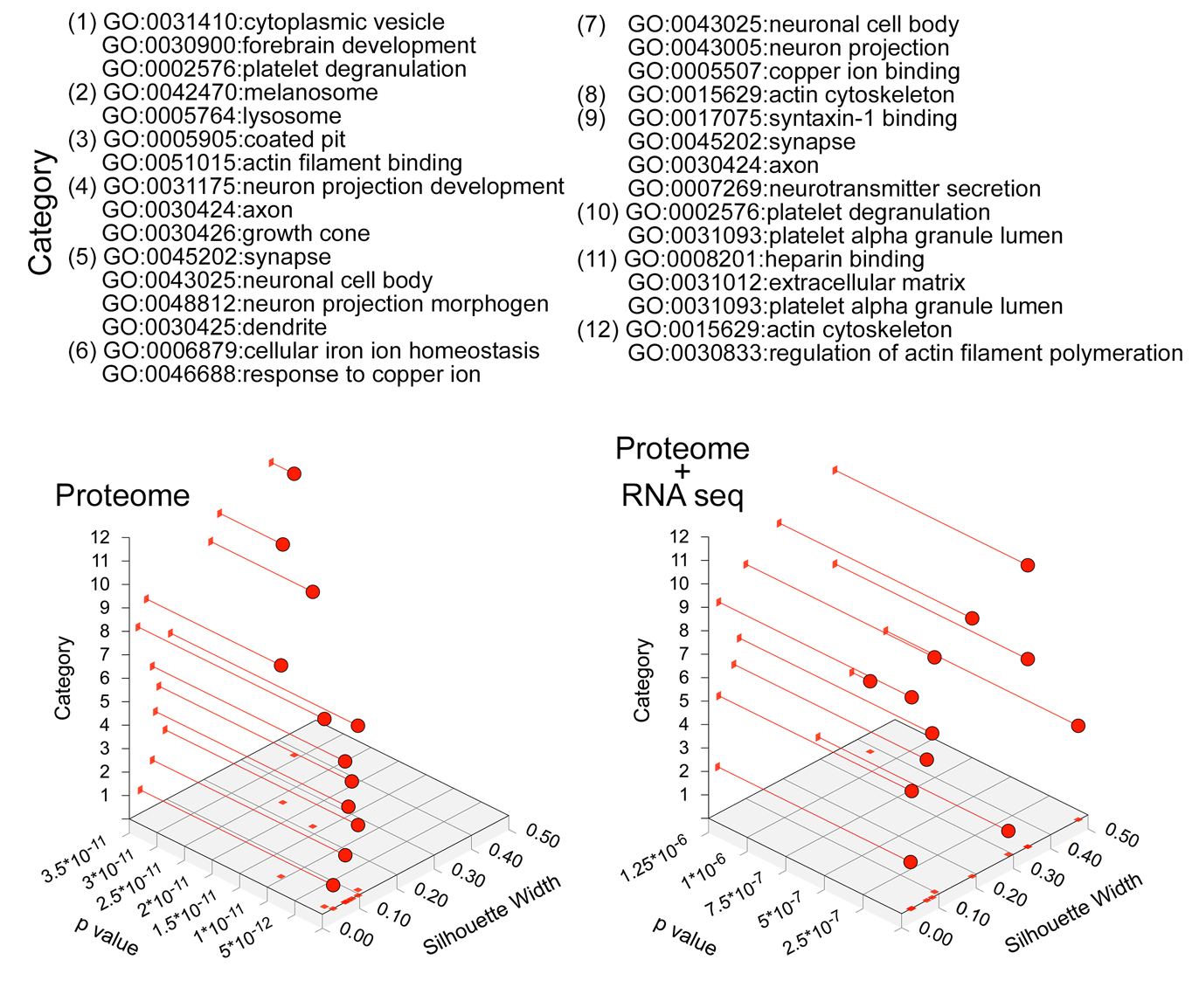

Supplement: FIGURE S1 — Ontology analysis of the BLOC-1 sensitive proteome with or without addition of the Bloc1s8sdy/sdy hippocampus transcriptome. Gene ontology analysis of the BLOC-1 sensitive proteome using the Geneterm Linker algorithm was performed with and without addition of the Bloc1s8sdy/sdy P7 hippocampus transcriptome hits. The numbers in the Z axis represent the gene ontology term groups defined by Geneterm Linker as metagroups (Fontanillo et al., 2011). The internal tightness of each metagroup and its separation from other metagroups is defined by the Silhouette score. Strong tightness between terms within a metagroup is defined by a positive Silhouette score. [file Image_1.TIF]
